# Whole fresh fruit intake and risk of incident diabetes in different glycemic stages: a nationwide prospective cohort investigation

**DOI:** 10.1007/s00394-022-02998-6

**Published:** 2022-10-19

**Authors:** Li Li, Hai-Yan Yang, Yan Ma, Xing-Huan Liang, Min Xu, Jie Zhang, Zhen-Xing Huang, Li-Heng Meng, Jia Zhou, Jing Xian, Ying-Jun Suo, Song Huang, Jin-Wei Cai, Bi-Hui Meng, Zhi-Yun Zhao, Jie-Li Lu, Yu Xu, Tian-Ge Wang, Mian Li, Yu-Hong Chen, Wei-Qing Wang, Yu-Fang Bi, Guang Ning, Fei-Xia Shen, Ru-Ying Hu, Gang Chen, Li Chen, Lu-Lu Chen, Hua-Cong Deng, Zheng-Nan Gao, Ya-Nan Huo, Qiang Li, Chao Liu, Yi-Ming Mu, Gui-Jun Qin, Li-Xin Shi, Qing Su, Qin Wan, Gui-Xia Wang, Shuang-Yuan Wang, You-Min Wang, Sheng-Li Wu, Yi-Ping Xu, Li Yan, Tao Yang, Zhen Ye, Xue-Feng Yu, Yin-Fei Zhang, Jia-Jun Zhao, Tian-Shu Zeng, Xu-Lei Tang, Ying-Fen Qin, Zuo-Jie Luo, 4C Study Group

**Affiliations:** 1 Department of Endocrinology, The First Affiliated Hospital of Guangxi Medical University, No. 6 of Shuangyong Road, Nanning, 530021 Guangxi China; 2 Department of Ultrasonography, The First Affiliated Hospital of Guangxi Medical University, Nanning, China; 3 Department of Endocrine and Metabolic Diseases, Shanghai Institute of Endocrine and Metabolic Diseases, Ruijin Hospital, Shanghai Jiao Tong University School of Medicine, Shanghai, China; 4 Shanghai National Clinical Research Center for Metabolic Diseases, Key Laboratory for Endocrine and Metabolic Diseases of the National Health Commission of the PR China, Shanghai National Center for Translational Medicine, Shanghai, China; 5 The First Affiliated Hospital of Wenzhou Medical University, Wenzhou, China; 6 Zhejiang Provincial Center for Disease Control and Prevention, Zhejiang, China; 7 Fujian Provincial Hospital, Fujian Medical University, Fuzhou, China; 8 Qilu Hospital of Shandong University, Jinan, China; 9 Union Hospital, Tongji Medical College, Huazhong University of Science and Technology, Wuhan, China; 10 The First Affiliated Hospital of Chongqing Medical University, Chongqing, China; 11 Dalian Municipal Central Hospital, Dalian, China; 12 Jiangxi Provincial People’s Hospital Affiliated to Nanchang University, Nanchang, China; 13 The Second Affiliated Hospital of Harbin Medical University, Harbin, China; 14 Jiangsu Province Hospital on Integration of Chinese and Western Medicine, Nanjing, China; 15 Chinese People’s Liberation Army General Hospital, Beijing, China; 16 The First Affiliated Hospital of Zhengzhou University, Zhengzhou, China; 17 Affiliated Hospital of Guiyang Medical College, Guiyang, China; 18 Xinhua Hospital Affiliated to Shanghai Jiao Tong University School of Medicine, Shanghai, China; 19 The Affiliated Hospital of Southwest Medical University, Luzhou, China; 20 The First Hospital of Jilin University, Changchun, China; 21 The First Affiliated Hospital of Anhui Medical University, Hefei, China; 22 Karamay Municipal People’s Hospital, Xinjiang, China; 23 Clinical Trials Center, Ruijin Hospital, Shanghai Jiao Tong University School of Medicine, Shanghai, China; 24 Sun Yat-Sen Memorial Hospital, Sun Yat-Sen University, Guangzhou, China; 25 The First Affiliated Hospital of Nanjing Medical University, Nanjing, China; 26 Tongji Hospital, Tongji Medical College, Huazhong University of Science and Technology, Wuhan, China; 27 Central Hospital of Shanghai Jiading District, Shanghai, China; 28 Shandong Provincial Hospital Affiliated to Shandong University, Jinan, China; 29 The First Hospital of Lanzhou University, Lanzhou, China

**Keywords:** Fruit consumption, Type 2 diabetes, Prediabetes, Normal glucose tolerance, Hazzard ratio

## Abstract

**Purpose:**

Fruit intake is beneficial to several chronic diseases, but controversial in diabetes. We aimed to investigate prospectively the associations of whole fresh fruit intake with risk of incident type 2 diabetes (T2D) in subjects with different glucose regulation capacities.

**Methods:**

The present study included 79,922 non-diabetic participants aged ≥ 40 years from an ongoing nationwide prospective cohort in China. Baseline fruit intake information was collected by a validated food frequency questionnaire. Plasma HbA1c, fasting and 2 h post-loading glucose levels were measured at both baseline and follow-up examinations. Cox proportional hazards models were used to calculate hazard ratio (HR) and 95% confidence intervals (CI) for incident diabetes among participants with normal glucose tolerance (NGT) and prediabetes, after adjusted for multiple confounders. Restricted cubic spline analysis was applied for dose–response relation.

**Results:**

During a median 3.8-year follow-up, 5886 (7.36%) participants developed diabetes. Overall, we identified a linear and dose-dependent inverse association between dietary whole fresh fruit intake and risk of incident T2D. Each 100 g/d higher fruit intake was associated with 2.8% lower risk of diabetes (HR 0.972, 95%CI [0.949–0.996], *P* = 0.0217), majorly benefiting NGT subjects with 15.2% lower risk (HR 0.848, 95%CI [0.766–0.940], *P* = 0.0017), while not significant in prediabetes (HR 0.981, 95%CI 0.957–4.005, *P* = 0.1268). Similarly, the inverse association was present in normoglycemia individuals with a 48.6% lower risk of diabetes when consuming fruits > 7 times/week comparing to those < 1 time/week (HR 0.514, 95% CI [0.368–0.948]), but not in prediabetes (HR 0.883, 95% CI [0.762–1.023]).

**Conclusion:**

These findings suggest that higher frequency and amount of fresh fruit intake may protect against incident T2D, especially in NGT, but not in prediabetes, highlighting the dietary recommendation of higher fresh fruit consumption to prevent T2D in normoglycemia population.

## Background

Type 2 diabetes (T2D) is a serious epidemic all over the world, which sees a great transition of lifestyles and dietary patterns during the last 3 decades [1]. The latest International Diabetes Foundation (IDF)’s global diabetes atlas (9th edition) shows that the global prevalence rate of diabetic adults is 9.3%, and is expected to be 10.9% in 2045 [2]. Overweight, obesity, imbalanced dietary habits and physical inactivity are modifiable among the major risk factors of T2D [3, 4]. Targeting the modifiable factors, such as the optimal selection of food and dietary intake, is supposed to be a promising way to curb the rise of T2D.

Dietary fresh fruit intake has been well established associated with beneficial effects on coronary heart diseases, stroke, some cancers, and related mortality [5, 6], due to its rich components of vitamin, potassium, dietary fiber and carotenoids [7]. Increasing fresh fruit intake, especially as a whole fruit, is recommended in dietary guidelines as an important part of healthy diet patterns for different regions and ethnics [8–10]. However, the association of fresh fruit consumption and risk of incident diabetes still lacked unified conclusion. Several prospective investigations showed no significant association between incidence of T2D with neither fruit only nor combined with vegetable intake [11, 12], while other meta-analysis and systematic reviews observed an inverse association between fruit consumption and risk of T2D [13–15]. Moreover, the effect of fruit intake on people in varied glucose metabolism states is poorly understood. Prediabetes is known to develop into T2D at distinct pace [16], and are the major reserve forces for diabetes. Particular dietary patterns, such as meat diet and fried food with staple diet (low in fresh fruit), were reported related to prediabetes [17]. Whether fruit consumption influences the progression from normoglycemia or prediabetes to T2D has not been investigated yet.

Therefore, we are interested in this present large nationwide prospective study to investigate the association of dietary consumption of fresh fruit, not only the frequency habits but also the semi-quantity consumption, with risk of incident T2D. The present study would provide more precise and prospective evidence in the prevention of diabetes for normal glucose tolerance (NGT) and prediabetes subjects by healthy eating diet rich in whole fresh fruit.

## Methods

### Design and study population

The study participants were from the ongoing China Cardiometabolic Disease and Cancer Cohort (4C) Study, which is a multicenter, population-based, prospective cohort study. The study design of the 4C Study has been described in detail previously [4, 18]. Briefly, at baseline, we recruited adults aged of 40 and above from local resident registration systems in 20 communities from various geographic regions in China in 2011–2012. All participants attended an in-person and on-site visit, and were invited to the first round of follow-up visit during year 2014 to 2016. Both at baseline and the follow-up, anthropometric and blood pressure measurements, oral glucose tolerance test (OGTT), and blood sampling were performed following a standard protocol. Demographic characteristics, lifestyle and dietary habits, and medical history were collected using standard questionnaires in face-to-face interview.

**Fig. 1:**
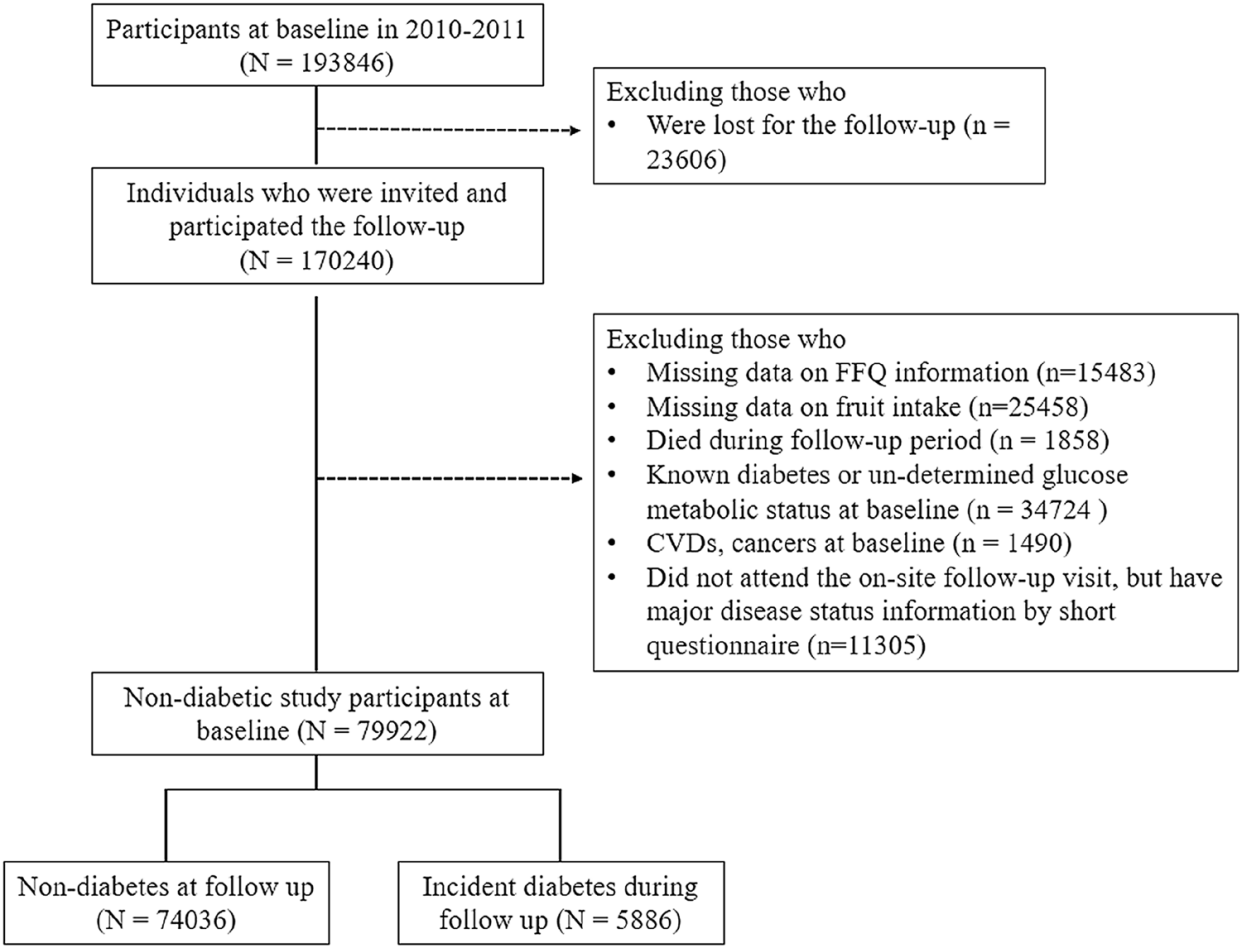
Study participants flow chart for the present study

There were 193,846 individuals participated the baseline examination and 170,240 (87.8%) finished the follow-up visits including a short questionnaire aiming to collect the information on major disease status and an on-site follow-up examination including blood sampling. For the present analysis, we further excluded those who were with missing data on the food frequency questionnaire (*n* = 15,843), failed to answer questions about fresh fruit consumption (*n* = 25,458), died during the follow-up period (*n* = 1858), with known diabetes or un-determined glucose metabolic status at baseline (*n* = 34,724), with cardiovascular diseases and cancers at baseline (*n* = 1490), or those who failed to attend the on-site follow-up examinations but had major disease status information by short questionnaire (without blood sampling at follow-up, *n* = 11,305). Finally, 79,922 non-diabetic individuals at baseline were included in the present analysis, and 5886 incident diabetes were ascertained during follow-up (Fig. 1).

This study was approved by the Medical Ethics Committee of Ruijin Hospital, Shanghai Jiaotong University. We obtained written informed consent from all participants.

### Data collection

Trained research personnel collected the data in local community clinics according to the standard process. In face-to-face questionnaire interviews, data on social demographic characteristics, education level, lifestyle habits (including physical activity, sedentary time, tobacco smoking and alcohol drinking habits), health status, and medical and family history were obtained. The physical activity information was accessed with International Physical Activity Questionnaire. Body mass index (BMI) was calculated as weight (kilograms) divided by height (squared meters). Blood pressure measurements were performed three times with validated automated electronic device (OMRON Model HEM-752 FUZZY) in a seating position after at least 5 min of rest. Fasting blood lipids, including total cholesterol, triglycerides, high-density lipoprotein cholesterol (HDL-C) and low-density lipoprotein cholesterol (LDL-C), were accessed using ARCHITECT ci16200 Chemistry Analyzer (Abbott Labs).

### The assessment of dietary fresh fruit intake

The dietary habits were collected at baseline by well-trained interviewers using a validated semi-quantitative food frequency questionnaire (FFQ), the validity of which has been evaluated in previous study [19]. The FFQ acquired the intake frequency and portion size of 21 food groups majorly consumed in Chinese population over the last 12 months, including cereal grains, root and tubers, meat (pork, beef, mutton), poultry (chicken, duck and goose), offal, fish and seafood, eggs, soybean products, milk products, vegetables, fresh fruit, fresh-made fruit or vegetable juice, fruit-flavored drinks, carbonated beverages, coffee, salted vegetables, pickled vegetables, fermented soybean curd, pastry, fried food, and nutrient supplement. For each food item, participants were prompted to report the average consumption as times per day, week, month, year or rarely/never, and the estimated quantity for corresponding frequency [20]. In the following analysis of fruit intake, all the responses were converted to daily frequency as four categories: less than once per week (< 1 time/week), 1–3 times/week, 4–7 times/week, and more than 7 times per week (> 7 times/week). The dietary intake of fresh fruit (gram/day, g/d) was based on the questionnaire, and was further assessed the association with risk of incident diabetes events by either each 100 g higher level or the quartiles increasing level.

### Ascertainment of the incident diabetes

OGTT and HbA1c were evaluated for all participants at baseline and follow-up. Blood was drawn in the morning after 8–12 h of fasting and 2 h after OGTT loading respectively. Fasting and 2 h plasma glucose (FPG and 2hPG) levels were measured with hexokinase or glucose oxidase method through a strict quality control process. HbA1c was assayed by high-performance liquid chromatography (VARIANT^™^ II Hemoglobin Testing System, Bio-Rad Laboratories) in central laboratory within 4 weeks after finger capillary whole-blood collection. T2D were defined basically according to the criteria with at least one of the following (1) FPG ≥ 7.0 mmol/L, or (2) OGTT 2hPG ≥ 11.1 mmol/L, or (3) one or more classic symptoms of diabetes and plasma glucose ≥ 11.1 mmol/L at any time on one day, or (4) HbA1c ≥ 6.5% (48 mmol/mol), or (5) a self-reported previous diagnosis of diabetes by physicians [21]. In this study, subjects with diabetes were excluded at baseline and the rest non-diabetic participants were further classified as normal glucose tolerance (NGT) or impaired glucose regulation (IGR) based on their glycemic variables. Ascertainment of incident T2D in the follow-up longitudinal analyses was a primary outcome. The NGT state was defined as FPG < 5.6 mmol/L and OGTT 2hPG < 7.8 mmol/L. Participants with IGR were also called prediabetes, referring to those with either impair fasting glucose (IFG, FPG 5.6–6.9 mmol/L, and OGTT 2hPG < 7.8 mmol/L), impaired glucose tolerance (IGT, FPG < 7.0 mmol/L and OGTT 2hPG 7.8–11.0 mmol/L) or combined IFG/IGT.

### Assessment of major lifestyle covariates

Information about multiple risk factors for diabetes or confounders was collected. The smoking or drinking status was coded as “yes” if the participant had smoked at least one piece of cigarette or consumed alcohol at least once a week in the last 6 months. Correspondingly, we divided the current smoking/drinking state into two categories: “smokers” (< 10 pieces/d, 11–19 pieces/d, and ≥ 20 pieces/d) and “non-smokers”, “drinkers” (< 20 g/d, 20–39 g/d, ≥ 40 g/d) and “non-drinkers”. Self-reported frequency (days per week) and the duration (minutes per day) of physical activity, as walking, mild, moderate, and vigorous activities were accessed, and average hours per day of activities at the above different intensity were calculated as the metabolic equivalent (MET)-hours per day. Education level was classified as “ < high school” or “ ≥ high school”. To control the influence of other dietary factors, we performed a principal component analysis (PCA) by taking all the other food intake information that was collected in the present study into account to construct the potential major dietary components as co-variates.

### Statistical analysis

Baseline characteristics of participants according to frequency of fresh fruit intake or baseline glucose metabolism status were summarized as means with standard deviations (SD), or medians with inter-quartile ranges for continuous variables, or numbers (proportions) for categorical variables. The variables with skewed distribution were log-transformed before statistical analysis. We also tested the homogeneity of the variance in groups and the population variance of each group is equal. Groups’ differences were tested using ANOVA test for continuous variables and chi-square for categorical variables.

Cumulative incidence (95% confidence interval, CI) of diabetes was calculated at average 3.8 years of follow-up. The multivariable Cox proportional hazards models were used to compute hazard ratios (HRs) and 95% CIs to estimate the relative risk of incident diabetes. Model 1 was adjusted for age, sex, study area; model 2 further adjusted for BMI and waist circumference based on model 1; model 3 further adjusted for physical activity, sedentary time, smoking and drinking status, education level, family history of diabetes, triglycerides, LDL-C and HDL-C based on model 2; model 4 further adjusted for other major dietary consumption components based on Model 3. We performed a principal component analysis (PCA) by taking all the other food intake information that was collected into account to construct the other major dietary components as co-variates. We used the “proc princomp” procedure in the SAS, using the TYPE option of CORR and without defining the number of the components.

We fixed the flexible regression models with restricted cubic spline (RCS) to examine the potential nonlinear associations between the dietary fruit intake (g/d) and the incidence of diabetes. We chose 15 g/day as the reference level (the lowest decile) and performed the RCS in total participants, and in NGT and prediabetes, separately as well. The knots were located at the 25th, 50th, and 75th percentiles for daily level of fresh fruit consumption. The adjustments were the same as above co-variables.

Analyses in the current study were performed by SAS version 9.4 (SAS Institute, Cary, NC). Statistical significance was considered at *P* value < 0.05 (two-tailed) for all tests.

## Results

### Baseline characteristics of participants

The baseline characteristics of participants according to fresh fruit consumption frequency are presented in Table 1. Of the 79,922 non-diabetic participants at baseline, the mean (SD) age was 55.40 (8.72) years, 32.64% were men, and 72.87% were IGR. Overall, 9.85% participants reported never or rarely consumed fresh fruit (for < 1 time/week), 30.68% for 1–3 times/week, 50.01% for 4–7 times/week, and the rest 9.46% for more than 7 times/week, respectively. Participants with less than once per week of fresh fruit consumption were with higher proportion in men, slightly thinner, less educated, more likely to smoke and drink, less physical activity, and with less diabetes family history than those with fruits intake > 7times/week (*P* < 0.0001).

**Table 1:** Baseline characteristics of participants according to frequencies of fresh fruit intake

Category	All	< 1 time/week	1–3 times/week	4–7 times/week	> 7 times/week
*n* (%)	79,922	7873 (9.85)	24,522 (30.68)	39,967 (50.01)	7560 (9.46)
Age, years	55.40 ± 8.72	55.98 ± 8.94	55.21 ± 8.83	55.37 ± 8.59	55.61 ± 8.75
Male sex, *n* (%)	26,084 (32.64)	3777 (47.97)	9446 (38.52)	10,857 (27.16)	2004 (26.51)
BMI, kg/m^2^	24.35 ± 3.48	24.15 ± 8.94	24.24 ± 3.44	24.39 ± 3.49	24.70 ± 3.58
Waist circumference, cm	83.28 ± 9.49	83.04 ± 9.66	83.00 ± 9.43	83.34 ± 9.47	84.09 ± 9.59
SBP, mmHg	129.19 ± 19.46	131.84 ± 19.99	130.08 ± 19.51	127.98 ± 19.12	129.95 ± 20.07
DBP, mmHg	77.29 ± 10.80	78.41 ± 10.97	77.57 ± 10.82	76.8 ± 10.67	77.75 ± 11.07
Smoking status, *n* (%)					
Non-smoker	68,113 (85.22)	5745 (72.97)	19,964 (81.41)	35,623 (89.13)	6781 (89.70)
Smoker < 10 pieces/d	1732 (2.17)	219 (2.78)	566 (2.31)	803 (2.01)	144 (1.90)
Smoker 11–19 pieces/d	3232 (4.04)	534 (6.78)	1210 (4.93)	1285 (3.22)	203 (2.69)
Smoker ≥ 20 pieces/d	6845 (8.56)	1375 (17.46)	2872 (11.34)	2256 (5.64)	432 (5.71)
Alcohol drinking status, *n* (%)					
Non-drinker	70,864 (88.67)	6157 (78.20)	21,181 (86.38)	36,605 (91.59)	6921 (91.55)
Drinker < 20 g/d	2029 (2.54)	261 (3.32)	654 (2.67)	938 (2.35)	176 (2.33)
Drinker 20–39 g/d	1918 (2.40)	312 (3.96)	678 (2.76)	777 (1.94)	151 (2.00)
Drinker ≥ 40 g/d	5111 (6.39)	1143 (14.52)	2009 (8.19)	1647 (4.12)	312 (4.13)
Physical activity, MET-hr/day	31.0 (0–46.2)	11.55 (0–46.2)	23.1 (0–16.2)	23.1 (8.25–46.2)	23.1 (3.3–46.2)
Sedentary time ≥ 4 h/day, *n* (%)	51,078 (64.63)	4287 (55.38)	15,072 (62.09)	26,890 (68.00)	4829 (64.68)
Education < high school, *n* (%)	46,451 (58.12)	5797 (73.63)	16,424 (66.98)	20,467 (51.21)	3763 (49.78)
Family diabetes history, *n* (%)	10,029 (12.55)	676 (8.59)	2541 (10.36)	5751 (14.39)	1061 (14.03)
Triglycerides, mmol/L	1.26 (0.90–1.81)	1.24 (0.88–1.80)	1.25 (0.90–1.81)	1.27 (0.91–1.81)	1.28 (0.92–1.82)
LDL-C, mmol/L	2.82 ± 0.86	2.72 ± 0.85	2.75 ± 0.84	2.87 ± 0.87	2.91 ± 0.88
HDL-C, mmol/L	1.33 ± 0.35	1.33 ± 0.36	1.32 ± 0.35	1.33 ± 0.35	1.34 ± 0.35
FBG, mmol/L	5.44 ± 0.53	5.46 ± 0.53	5.43 ± 0.52	5.43 ± 0.53	5.46 ± 0.51
2hPBG, mmol/L	6.81 ± 1.64	6.75 ± 1.68	6.75 ± 1.65	6.85 ± 1.63	6.84 ± 1.64
HbA1c, % in NGSP value	5.69 ± 0.38	5.68 ± 0.28	5.67 ± 0.38	5.71 ± 0.38	5.70 ± 0.38
HbA1c, mmol/mol	38.72 ± 4.17	38.60 ± 4.18	38.46 ± 4.19	38.89 ± 4.17	38.81 ± 4.11

Continues variables are presented as means ± standard deviation (SD), or medians (inter-quartile ranges) for skewed variables, or number (proportions) for categorical variables

*BMI *body mass index; *SBP* systolic blood pressure; *DBP* diastolic blood pressure; *MET* metabolic equivalent task; *LDL-C* low-density lipoprotein cholesterol; *HDL-C* high-density lipoprotein cholesterol; *FBG* fasting blood glucose; 2 h *PBG* 2 h post OGTT loading blood glucose; *HbA1c* hemoglobin A1c. HbA1c (mmol/mol) = [HbA1c (% in NGSP value) − 2.15] × 10.929

### Frequency of fresh fruit intake and risk of incident diabetes

The cumulative incident rate of diabetes was lower in the higher fruit intake frequency groups (*P* = 0.0013, Table 2). The Cox regression analysis showed that fruit intake frequency was significantly and inversely associated with risk of incident diabetes. As compared to those consumed fresh fruit < 1 time/week, subjects consumed for 1–3 times/week, 4–7 times/week and > 7 times/week were associated with 1.7% (HR 0.983, 95%CI [0.898–1.077], 8.1% (HR 0.919, 95%CI [0.840–1.005], and 16.4% (HR 0.836, 95% CI [0.741–0.943]) lower risk of incident diabetes, respectively (*P* for trend = 0.0042, Model 3), after adjusting for the traditional risk factors of diabetes including age, sex, BMI, waist circumference, physical activity, sedentary time, smoking and drinking status, education level, family history of diabetes, triglycerides, LDL-C and HDL-C. Additional adjustment for other major dietary consumption components did not materially alter such association (Model 4 in Table 2). Frequent fresh fruit consumers (> 7 times/week) were associated with 15.8% decreased risk of diabetes, as compared to those consumed fresh fruit < 1 time/week (*P* = 0.0106, Model 4), but not in other frequencies as 1–3 times/week or 4–7 times/week [adjusted HR, (95%CI): 0.973(0.878–1.080), *P* = 0.6100; 0.924(0.833–1.024), *P* = 0.1307, respectively].

**Table 2:** Incidence rate and risk of incident diabetes associated with frequency of fresh fruit consumption

	< 1 time/week	1–3 times/week	4–7 times/week	> 7 times/week	*P* trend
Incident diabetes, *n* (%)*	630 (8.00)	1842 (7.51)	2909 (7.28)	505 (6.68)	
Model 1	Ref. (1.00)	1.00 (0.91–1.09)	0.96 (0.88–1.05)	0.87 (0.77–0.98)	0.0407
	*p*	0.9910	0.3650	0.0199	
Model 2	Ref. (1.00)	0.99 (0.90–1.08)	0.94 (0.86–1.03)	0.84 (0.75–0.95)	0.0095
	*p*	0. 7945	0.1796	0.0044	
Model 3	Ref. (1.00)	0.98 (0.90–1.08)	0.92 (0.84–1.01)	0.84 (0.74–0.94)	0.0042
	*p*	0.7195	0.0632	0.0035	
Model 4	Ref. (1.00)	0.97 (0.88–1.08)	0.92 (0.83–1.02)	0.84 (0.73–0.97)	0.0527
	*p*	0.6100	0.1307	0.0106	

Data are hazard ratio (HR), 95% confidence interval (CI). P value for trend was for the Wald statistic, the “type III” results, for the additive model from the multivariable Cox regression models. *P* values were from the maximum likelihood estimate analysis. Model 1, adjusted for age, sex, and study areas; Model 2, adjusted for age, sex, BMI, waist circumference; Model 3, adjusted for age, sex, BMI, waist circumference, physical activity, sedentary time, smoking and drinking status, education level, family history of diabetes, triglycerides, LDL-C and HDL-C; Model 4, further adjusted for other major dietary consumption components based on Model 3. Total *n* = 79,922. * as *P* for Mantel–Haenszel chi-square test to compare incident rate of diabetes among the four groups was 0.0013. *p* value was for the risk of diabetes in relation to each specific group of fruit intake, as compared to the < 1time/week

### Amount of fruit intake and risk of incident diabetes

We assessed the association between risk of incident diabetes and total daily servings of fresh fruit (grams per day, g/d) calculated based on the self-reported frequency and quantity (grams/times) for each time of consumption. The average fruit consumption in our study was 100 (28.7–150) g/d. Meanwhile, only 4540 (5.98%) of the total participants reached the recommended standards of more than 300 g/d. In the categorical analyses according to quartiles of calculated total daily intake of fruit, compared to those whose consumption < 28.6 g/d, higher consumed groups in 28.7–99 g/d, 100–149 g/d, and ≥ 150 g/d were associated with gradually decreased risk of incident diabetes with adjusted HRs as 0.922 (95% CI 0.855–0.993), 0.915 (0.845–0.992), and 0.875 (0.809–0.946), respectively (*P* for trend = 0.0093, Model 3 in Table 3), independent of age, sex, BMI, waist circumference, physical activity, sedentary time, smoking and drinking status, education level, family history of diabetes, triglycerides, LDL and HDL levels. This trend persisted after further adjustment for other major dietary consumption components on base of Model 3 (Model 4 in Table 3, *P* for trend = 0.0375). Moreover, each 300 g/d higher intake of fresh fruit was associated 8.2% lower risk of incident diabetes (HR 0.918, 95%CI [0.853–0.987], *P* = 0.0207) after adjusted confounders in Model 4. Each 100 g/d higher fruit intake was associated with 2.8% lower risk of diabetes [HR (95%CI): 0.972, (0.949–0.996)], majorly benefiting NGT subjects with 15.2% lower risk (*P* = 0.0017), while not significant in prediabetes (HR 0.981, 95%CI 0.957–4.005, *P* = 0.1268).

**Table 3:** Incidence rate and risk of incident diabetes associated with fresh fruit consumption on a daily intake level

	Fresh fruit intake (g/d)				
	< 28.6	28.7–99	100–149	≥ 150	*P* trend
Incident diabetes, *n* (%)*	1518 (7.72)	1183 (6.98)	1352 (7.63)	1500 (6.95)	
HR (95% CI)					
Model 1	Ref. (1.00)	0.92 (0.86–0.99)	0.94 (0.87–1.02)	0.915 (0.85–0.99)	0.0778
	*p*	0.0272	0.1161	0.0196	
Model 2	Ref. (1.00)	0.92 (0.85–0.99)	0.93 (0.86–1.01)	0.90 (0.83–0.97)	0.0311
	*p*	0.02176	0.0657	0.0050	
Model 3	Ref. (1.00)	0.92 (0.86–1.00)	0.915 (0.85–0.99)	0.875 (0.81–0.95)	0.0093
	*p*	0.0331	0.0302	0.0008	
Model 4	Ref. (1.00)	0.90 (0.83–0.99)	0.912 (0.84–1.00)	0.888 (0.81–0.97)	0.0375
	*p*	0.0229	0.0394	0.0073	

The categorical analyses were according to quartiles of calculated total daily intake of fruit. Data are hazard ratio (HR) and 95% confidence interval (CI). *P* values for trend was for the Wald statistic, the “type III” results, for the additive model from the multivariable Cox regression models. Model 1, adjusted for age, sex, and study areas; Model 2, adjusted for age, sex, BMI, waist circumference; Model 3, adjusted for all covariates in Model 2 plus physical activity, sedentary time, smoking and drinking status, education level, family history of diabetes, systolic and diastolic blood pressure, triglycerides, LDL-C and HDL-C; Model 4, further adjusted for the major dietary consumption components based on Model 3. Total *n* = 75,917 (*n* = 4005 were missing for the information on fruit consumption on a daily intake level). * as *P* for Mantel–Haenszel chi-square test was 0.0290. *p* value was for the risk of diabetes in relation to each specific group of fruit intake, as compared to the < 28.6 g group

**Fig. 2:**
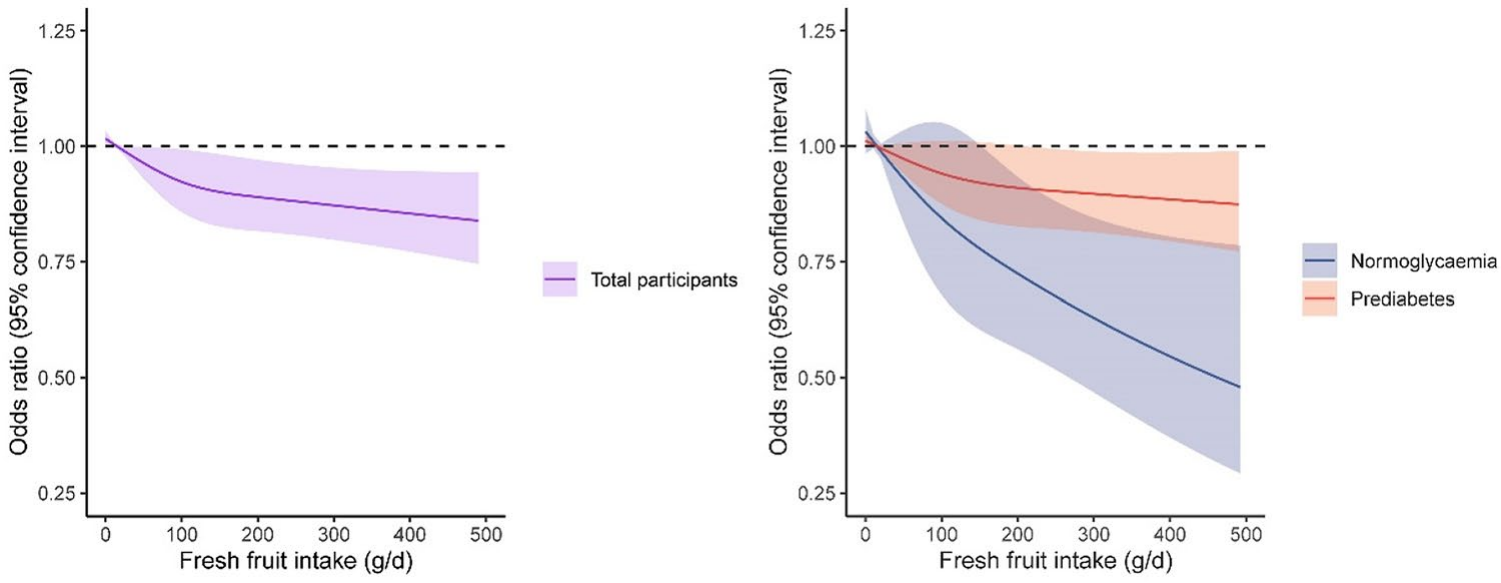
The cubic spline analysis of the association of incident diabetes with daily fresh fruit intake. The solid lines indicate multivariate adjusted odds ratios and the dashed lines indicate the 95% confidence intervals derived from the restricted cubic spline regression. A knot is located at the 25th, 50th, and 75th percentiles for daily level of fresh fruit intake. The Logistic regression model was adjusted for age, sex, study area, BMI, waist circumference, family history of diabetes, smoking, drinking, education status, physical activity, systolic blood pressure, HDL-C, LDL-C, and triglycerides, and other major dietary consumption components. *P* for lin = 0.0363, and *P* for non_lin_association = 0.1213 in total participants. *P* for lin = 0.1988, and *P* for non_lin_association = 0.6981 in prediabetes participants. *P* for lin = 0.0864, and *P* for non_lin_association = 0.1812 in normal glucose participants

Additionally, multivariable-adjusted restricted cubic spline analyses suggested a significant linear relationship between daily fresh fruit intake and incident diabetes (*P* for lin = 0.0363, and *P* for non_lin_association = 0.1213 in total participants, Fig. 2).

### Stratified analysis of the association of fruit intake with risk of diabetes

**Fig. 3:**
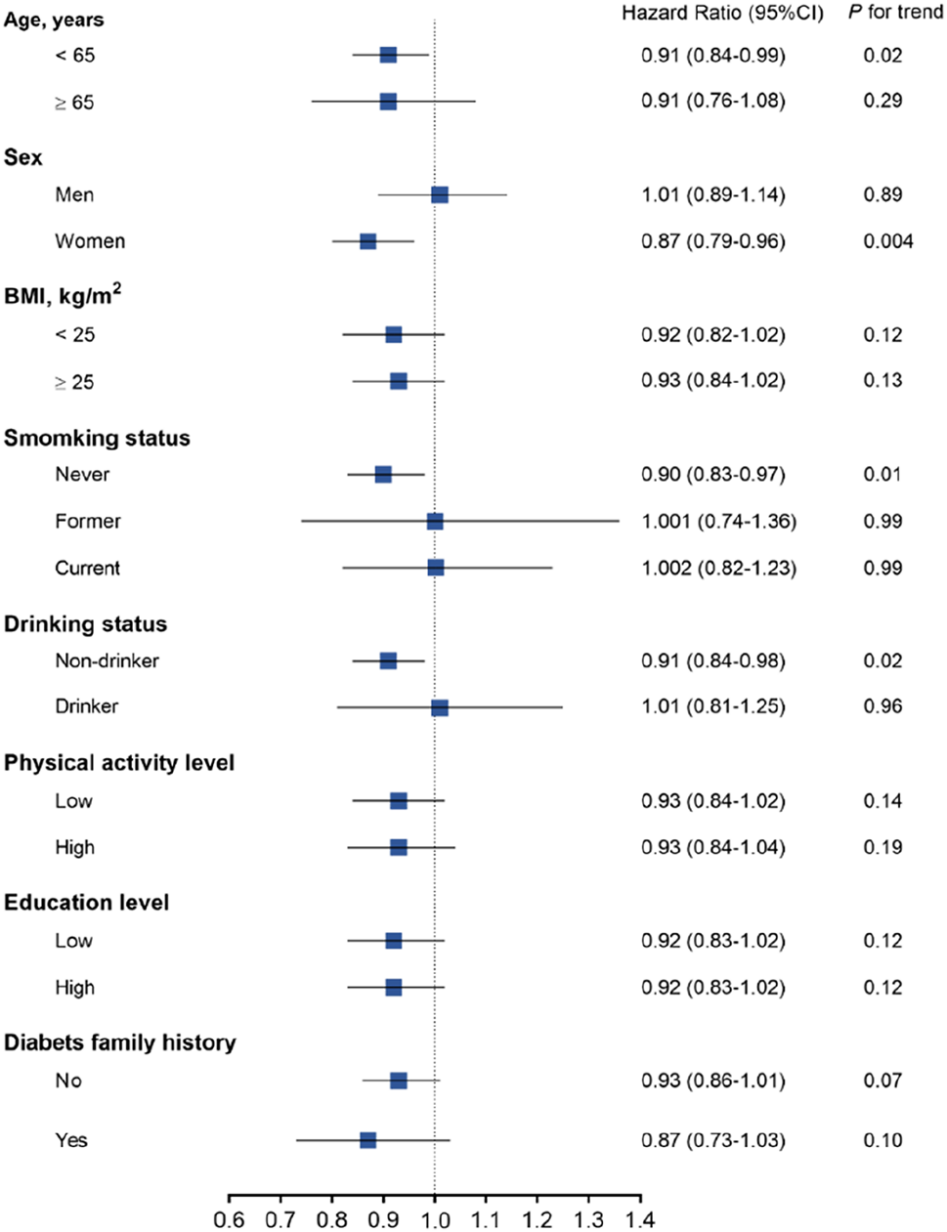
Stratified analysis of the association of fruit intake with risk of diabetes. Data are hazard ratio (HR), 95% confidence interval (CI). The Cox regression models were fitted with each 300 g intake of fruit intake with risk of incident diabetes, after adjustments for age, sex, BMI, waist circumference, physical activity, sedentary time, smoking and drinking status, education level, family history of diabetes, triglycerides, LDL-C, HDL-C

Subsequent analyses were then conducted stratified according to glucose metabolism status at baseline as NGT group and IGR group (or prediabetes). The inverse association found between the frequency of fresh fruit consumption and the risk of incident diabetes was significant in NGT population (*P* = 0.0140), yet not in IGR group (*P* = 0.2764) (Model 2 in Table 4). Furthermore, subgroup with fruit consumption > 7 times/week in NGT-baseline population had almost 50% lower risk of diabetes (adjusted HR 0.514, [95%CI 0.368–0.948], *P* = 0.0292, Model 2) verse subgroup of < 1 time/week, but also not significant in IGR-baseline subjects (adjusted HR 0.883, [95%CI 0.762–1.023, *P* = 0.0963, Model 2). Such protective effect of diabetes was not demonstrated in other two frequencies of 1–3 times/week and 4–7 times/week for both NGT and IGR (Model 2). Regarding daily fruit consumption, per 300 g/d fruit intake was associated with 40.2% decreased risk of incident diabetes (95% CI 0.432–0.828, *P* = 0.0019) in NGT population, but not in the IGR population (HR 0.950, 95% CI [0.881–1.026], *P* = 0.1897). Moreover, 4379 (20.8%) NGT (*n* = 21,031) at baseline developed prediabetes. We assessed the association of each 100 g/day intake of fresh fruit with risk of developing prediabetes, and found the odds ratio (95% CI) was 0.954 (0.924 0.985) (*P* = 0.0042) from the multivariable logistic regression analysis multivariable-adjusted. Stratified analysis also showed that fruit intake was significantly associated with lower risk of type 2 diabetes in age less than 65 years, in women, never smokers, and non-drinkers (Fig. 3).

**Table 4:** Risk of incident diabetes associated with frequency of fresh fruit consumption by baseline glucose metabolism

	< 1 time/week	1–3 times/week	4–7 times/week	> 7 times/week	*P* trend
Normal glucose tolerance (*n* = 21,679)					
*n*. cases/participant	**65/2094**	**217/7001**	**317/10,584**	**49/2000**	
Model 1	Ref. (1.00)	1.08 (0.82–1.42)	0.97 (0.73–1.27)	0.67 (0.46–0.97)	0.0308
	*p*	0.6013	0.8000	0.0360	
Model 2	Ref. (1.00)	1.14 (0.83–1.57)	1.02 (0.74–1.42)	0.51 (0.37–0.95)	0.0140
	*p*	0.4191	0.8860	0.0292	
Impaired glucose regulation (*n* = 58,243)					
*n*. cases/participants	**565/5779**	**1625/17,521**	**2592/29,383**	**456/5560**	
Model 1	Ref. (1.00)	1.00 (0.90–1.10)	0.96 (0.87–1.05)	0.89 (0.79–1.01)	0.1645
	*p*	0.9750	0.3883	0.0722	
Model 2	Ref. (1.00)	0.97 (0.87–1.08)	0.92 (0.83–1.03)	0.88 (0.76–1.02)	0.2764
	*p*	0.5332	0.1525	0.0963	

Data are hazard ratio (HR), 95% confidence interval (CI). *P* values for trend was for the Wald statistic, the “type III” results, for the additive model from the multivariable Cox regression models. Model 1, adjusted for age, sex, and study areas; Model 2, further adjusted for BMI, waist circumference, physical activity, sedentary time, smoking and drinking status, education level, family history of diabetes, systolic and diastolic blood pressure, triglycerides, LDL-C, HDL-C, and other major dietary consumption components, based on Model 1. *p* value was for the risk of diabetes in relation to each specific group of fruit intake, as compared to the < 1 time/week

Bold values descriptive for case number in each groups and need not to be compared, thus do not have *P* values for them

## Discussion

In this nationwide prospective cohort study of Chinese adults aged 40 and above, a linear and dose-dependent inverse association was found between daily whole fresh fruit intake and incident diabetes. This inverse association was present in normoglycemia individuals, while not markedly significant in the prediabetes counterparts. To the best of our knowledge, this study is one of the first to reveal the different risk impact of fruit intake on diabetes among people with varied glycemia modulating abilities assessed by fasting and 2 h post-loading blood glucose and the HbA1c levels.

Previous studies investigating the association between fruit intake and the risk of T2D were inconsistent and even controversial. Several cohort studies and meta-analysis showed no relation between fruit intake and diabetes [22, 23]. However, a previous large sample Chinese cohort, the China Kadoorie Biobank (CKB) study, showed that higher fruit consumption was significantly associated with lower risk of developing diabetes for those without prevalent diabetes, and with reduced risk of overall death and major vascular complications for those already diagnosed of diabetes [21]. In addition, people with low or inadequate fruit servings were reported more likely to experience T2D [24] and gestational diabetes mellitus [25]. Reasons for the inconsistent findings include various sample sizes, different evaluation methods for food consumption, and the heterogeneity across populations. Our findings, based on nationwide prospective study design, are in line with prior high-quality prospective studies, suggesting that more whole fresh fruit intake was associated with lower diabetes incidence.

The potential mechanism underlying the protective effect of fruit intake in delaying or preventing T2D development may lie in the whole fruits’ rich sources of fibers, flavonoids, and various antioxidant compounds [26, 27]. Investigation on fruit juice has shown a higher risk association with diabetes [28], thereby suggesting that the relative lack of fiber and the liquid state of fruit juice may be harmful in diabetes prevention. The fiber and other positive substances in the whole fruit can modulate molecular pathways in immunological reactions and thus activate immune system [29], and contribute to the gut flora diversity and other colonic microbial population composition [30]. Phytochemicals and their metabolic products could inhibit disease-causing bacteria and stimulate the helpful types, exerting prebiotic-like effects [31]. High fruit intake was potentially beneficial for human health through increasing production of short-chain fatty acids, maintaining intestinal mucosal integrity, and improving insulin sensitivity and anti-inflammatory properties [32]. In addition, fruit and the contained phytochemicals and vitamins may play an important role in modulating chronic disease risk through certain gene or DNA methylation [33, 34]. Our recent study [19] was broadly consistent with relevant research [35], suggesting fruit intake was interacting with genetic predisposition of T2D on the risk of diabetes, and phytonutrients may affect genes involved in insulin synthesis or insulin resistance, oxidative stress, stimulus-secretion coupling, anti-glucolipotoxicity, and inflammation [36, 37], which in turn could explain the inconsistent study conclusions from different populations.

Another noteworthy discovery in our study is the significant decrease in risk of diabetes with fruit intake in NGT population, but not significant in established prediabetes. That contrasts with the findings from several prospective cohorts, showing that dietary antioxidant capacity [38] and fiber [39] derived from fruit were inversely associated with risk of diabetes in prediabetes subjects, and risk of prediabetes in normal people, respectively. In a RCT to prevent diabetes in overweight/obese prediabetes individuals, lifestyle intervention including education and support could reduce risk of diabetes with HR of 0.49 at one year, among which 12.2% of the risk fall attributed to the change of increasing fruit and vegetable intake [40]. Therefore, we could not roughly conclude that fresh fruit was of no benefit on lowering the risk of diabetes in prediabetes subjects. But a public general recommendation for all people including prediabetes for more fruit intake to prevent diabetes should be cautious, based on the results of our large sample prospective cohort study. As to the reason of absent significant risk reduction in prediabetes in our study, the more serious declined self-regulation ability to blood glucose in prediabetes people should be considered. Besides, other more overwhelming factors, such as genetic background, baseline risk and other life habits, could attenuate the benefit of fruit in diabetes prevention in prediabetes population. In our study, the incident diabetes exhibited more likely were male, current smoker or drinker, with higher BMI, family history of diabetes, higher blood pressure and lipid disorder. The stratified analysis suggested that higher consumption of fruit seems to be protective for the onset of T2D in women, but not in men. This could be due to the combined presence of synergistic risk factors, such as low fruit intake proportion, drinking and smoking status, and higher systolic blood pressure, which is more prevalent in men than in women in our data.

The non-statistically significant decreased risk of diabetes in subgroups of 1–3 times/week and 4–7 times/week, comparing to group of < 1 time/week, for both NGT and prediabetes could be attributed partly to the inadequate dietary circulating positive properties of accumulative fruit intake. Our data also demonstrated a surprising general insufficient daily fruit intake in people of non-diabetes over 40 years old in China. Only 9.46% of the total subjects reached the frequency of > 7 times/week, a frequency which achieving significant risk-reduced effect of diabetes when compared to the rarely consumers. Meanwhile, only 5.98% of the total participants reached the recommended standards of more than 300 g/d. Along with the inverse association between fruit intake and diabetes found in our study with the previous ones, we speculated that the insufficient fruit intake in senior Chinese citizen over 40 years old may has certain relationship with the overall rise of diabetes in the recent years.

The strengths of our study included the large sample size, prospective longitudinal and nationwide multicenter design, use of representative dietary habits for different areas of China, the standard study protocol, and the relatively comprehensive information collection on demographic, lifestyle, and other covariates, thus minimizing confounding bias. We were also able to attain the reliable centralized HbA1c and glucose-related metabolic phenotypes data at baseline and follow-up, which reflect the stable glucose regulation and enable us to reach a more accurate conclusion in the stratified analysis for NGT and prediabetes. However, the primary limitation was that we did not obtain detailed types of fruit consumed, which may highly diverse in contents of fiber and glycemic index (GI) that jointly influenced the risk. Previous studies have demonstrated both high and low GI fruit associated with lower risk of diabetes [41], though other investigation also asserted that lower GI (i.e., apples, pears, oranges, and berries) may have larger benefits on diabetes prevention [42]. According to the China Health and Nutrition Survey, the most frequently consumed fruits for Chinese population are temperate fruit with low GI, like apples, pears, and oranges. Second, although we have adjusted for relative comprehensive confounders, the unmeasured residual confounding factors like other dietary influence are still potentially existing. Finally, fruit consumption was assessed with the semi-quantitative food frequency questionnaire of FFQ, which is based on perceptions of habitual intake and may result in potential recall bias.

## Conclusion

In conclusion, higher fruit intake frequency and amount were associated with lower incidence of T2D in Chinese adults over 40 years old. The most prominent risk reduction of diabetes was in subjects consuming whole fresh fruit at least 7 times/week. Each 300 g/day higher fruit intake was associated with an 8.2% lower risk of diabetes, and each 100 g/day higher fruit intake was similarly associated with 2.8% lower risk of diabetes. The negative linear association between fruit intake and incident diabetes was markedly in NGT population, while not significant in prediabetes. These findings emphasize the importance and effect of dietary whole fresh fruit, at least more than 7 times/week especially in NGT population, in diabetes prevention. Our data are promising to provide concrete social and clinical guidance in public health education to reduce diabetes events with consideration of different glycemic stages to adopt different fruit diet recommendations.

## Data Availability

Data are available on request to corresponding author.
